# Benign paroxysmal positional vertigo recurrence and persistence

**DOI:** 10.1016/S1808-8694(15)30497-3

**Published:** 2015-10-19

**Authors:** Ricardo S Dorigueto, Karen R Mazzetti, Yeda Pereira L Gabilan, Fernando Freitas Ganança

**Affiliations:** 1 Otorhinolaryngologist. MSc and PhD student in Sciences - Graduate Program in Otorhinolaryngology and Head and Neck Surgery - UNIFESP-EPM; 2 Physical therapist. Specialist in Exercise Physiology - FMUSP- IOT/HC; 3 Physical therapist. MSc and PhD student in Sciences - Graduate Program in Otorhinolaryngology and Head and Neck Surgery - UNIFESP-EPM. Professor of UNICID; 4 Otorhinolaryngologist, PhD in Medicine - UNIFESP - EPM. Adjunct Professor of Neurotology - UNIFESP - EPM. Professor at the Graduate Program in Vestibular Rehabilitation and Social Inclusion - UNIBAN

**Keywords:** vestibular diseases, pathologic nystagmus, rehabilitation, vertigo

## Abstract

Benign paroxysmal positional vertigo (BPPV) is one of the most common vestibular disorders.

**Aim:**

To study the recurrence and persistence of BPPV in patients treated with canalith repositioning maneuvers (CRM) during the period of one year.

**Study design:**

longitudinal contemporary cohort series.

**Materials and Methods:**

One hundred patients with BPPV were followed up during 12 months after a treatment with CRM. Patients were classified according to disease evolution. Aquatic physiotherapy for vestibular rehabilitation (APVR) protocol was applied in cases of persistent BPPV.

**Results:**

After CRM, 96% of the patients were free from BPPV's typical nystagmus and dizziness. During the follow up period of 1 year, 26 patients returned with typical BPPV nystagmus and vertigo. Nystagmus and vertigo were persistent in 4% of the patients. Persistent BPPV presented improvement when submitted to APVR. Conclusion: During the period of one year, BPPV was not recurrent in 70% of the patients, recurrent in 26% and persistent in 4%.

## INTRODUCTION

Benign paroxysmal positional vertigo (BPPV) is characterized by rotatory dizziness triggered by head movements such as the ones caused by neck hyperextension and when the patient stands up or lies down on bed^1^.

Vertigo and other associated symptoms are triggered by fragments of statocones coming from the utriculus macula, which move to one or more semicircular canals and turn the cupule into a gravity-sensitive organ^2^.

A number of treatments have been proposed for BPPV, including drugs, surgery and vestibular rehabilitation exercises. Cupula lithiasis and duct lithiasis theories allowed for the creation of maneuvers aiming at cleaning the cupule and the semicircular canal ducts of statocone fragments. In 1988, in Paris, Alain Semont et al.^3^ and in 1992, in the USA, Epley^4^ described the first statocone repositioning maneuvers (SRM). According to them, the success rate after one session was of 83.96% for the Semont maneuver and 97.70% for the Epley's.

Despite the great efficacy of SRMs and the possible spontaneous resolution, in 20 to 30% of the patients the disease can recur or persist^5^,^6^. In cases of BPPV recurrence, maneuver repetition is useful to shorten the duration of dizziness. Nonetheless, the persistent form does not respond to SRM treatment, and additional strategies may be necessary - such as vestibular rehabilitation exercises, vestibular function suppressive drugs and surgical procedures^7^.

Many authors stress the benefits of vestibular rehabilitation exercises on the ground in relation to the improvement of balance and vestibular compensantion^8^, ^9^, ^10^, ^11^, ^12^, ^13^, ^14^.

Gabilan15 and Gabilan et al.^16^ developed an aquatic physical therapy protocol as a method for vestibular rehabilitation (APVR) for patients with vestibular balance disorders and noticed that patients with unilateral deficitary peripheral vestibular syndrome (UDPVS), characterized by hypo or hyperreflexia seen at the caloric test had improvements in their self-perception of dizziness, quality of life and performance at the computerized dynamic posturography when assessed before and after such treatment.

The goal of the present investigation was to describe BPPV recurrence rate and persistence within a one year period.

## METHODS

This study was approved by the Ethics in Research Committee of the University where it was held, under protocol # 0596/04.

This is a longitudinal cohort study, in which one hundred consecutive patients with BPPV diagnosis were included.

The patients were submitted to neurotological evaluation including interview, otolaryngological evaluation, threshold tonal audiometry, vocal discrimination test, impedance measures, static and dynamic balance studies and computerized electronystagmography (ENG).

The patients included in the study had positional nystagmus and vertigo which are characteristic of BPPV. BPPV is characterized by recurrent spells of rotational vertigo during seconds, triggered by head movements, and it can happen with nausea, vomit, paleness and sweating. The characteristics of positional nystagmus indicate the labyrinth (s) and the semicircular canal (s) damaged, making the distinction between duct lithiasis (nystagmus lasting for less than one minute) and cupule lithiasis (nystagmus lasting for more than one minute). Vertical positional nystagmus upwards and counter-clockwise or clockwise rotational characterize the involvement of the right and left posterior semicircular canal, respectively; positional nystagmus downwards and counter-clockwise or clockwise rotational characterize the involvement of the right and left anterior canal, respectively; exclusively clockwise or counter-clockwise rotational nystagmus indicates involvement of one of the vertical right or left canal, respectively; and horizontal positional nystagmus characterizes involvement of the lateral canal.

In order to study the positional nystagmus we performed a Dix Hallpike maneuver^17^ with Frenzel goggles, starting in the position which triggered the vertigo and/or nystagmus, according to the information obtained from each patient. When the patient was unable to report which position was responsible for triggering the vertigo spell, the maneuver started with the right side. The patients were classified according to the pathophysiological substrate and the semicircular canal involved, indicated by the nystagmus characteristics and the triggering movement, as described by Herdman^18^.

We took off the study those cases of positional dizziness with atypical nystagmus, that is, those with different characteristics than the ones described by Herdman18 during the diagnostic maneuver.

All the patients were weekly treated by means of SRMs according to the pathophysiological substrate and the semicircular canal involved until the positional vertigo and nystagmus subsided. For the posterior canal we used the modified Epley Maneuver^19^. For the lateral canal we used the Lempert maneuver^20^. For the anterior canal we also used the modified Epley maneuver, as described by Herdman^21^.

Patients were classified in three groups, according to clinical evolution after SRM and one year clinical follow up:

1) Non-recurrent BPPV;
2) recurrent and
3) persistent.

Thus, we considered non-recurrent BPPV those patients who did not have BPPV recurrences during this period, after SRM. We considered it recurrent BPPV the clinical manifestation of vertigo signs and symptoms after vertigo and nystagmus subsiding with SRM, seen by the otorhinolaryngologist along one year after SRM. These patients became symptomatic again after SRM repetition. The BPPV persistent group was that group of patients in which there was no nystagmus or vertigo improvement with the weekly performance of SRM for at least 10 sessions monitored by the otorhinolaryngologist and, after this period, by means of a daily self-treatment with the Epley maneuver done at home^4^.

The patients were followed up by means of weekly consultations during the treatment period with SRMs, and monthly during follow up of the persistent cases.

The patients who persisted with positional vertigo and nystagmus characteristics of BPPV were submitted to the APVR protocol after neurological evaluation, brainstem audiometry and brain MRI with normal results.

The APVR protocol is made up of 12 stages of exercises and applied in 10 sessions of 45 minutes each, thrice a week, without home exercises, with mean duration of one month. The sessions were held in a warm pool, with temperature varying between 33.8 and 34.2° C, controlled by a thermostat. The pool depth was 1.3m in the shallowest area and 1.5m in the deepest one. In one of the pool walls there was a water whirling system with a motor. Access to the pool was by masonry steps, coated by anti-slippery floor, with 10cm-high steps. APVR is divided into twelve stages: water adaptation; turning off; postural transference; trunk rotational control; trunk rotational control associated with mobile target tracking; thrust gait; going up and down steps; seated flotation; seated flotation associated with mobile target tracking; standing flotation; standing flotation associated with mobile target tracking, and movement control with maximum whirl^16^.

Prior to treatment, up to one week before and after it, patients with persistent BPPV were evaluated by means of a Brazilian DHI quality of life questionnaire, analogue dizziness scale and balance tests (Romberg, Sensitized Romberg and unipodal support).

The Brazilian DHI^22^ assesses the loss stemming from dizziness in the quality of life of the patients, in relation to the physical, functional and emotional aspects and total score, being made up of twenty five questions in which the patient can chose among the following answers: yes, no, and sometimes. The “Yes” answer yields four points. “Sometimes” yields 2 points; and “No” gets zero point. The score goes from 0 to 100. One hundred points indicate the most impact on quality of life because of dizziness. By the same token, the lower the score, the lower is its impact on the patient's quality of life.

The dizziness analogue measures the patient's self perception of his/her dizziness intensity at the time of the evaluation, which can yield 0 to 10 points, 0 meaning no dizziness and 10 meaning maximum dizziness.

Patient balance was assessed by means of the Romberg test in the following sensorial conditions:

1) Romberg with eyes open, biped support in a firm surface;
2) Romberg with eyes open, biped support in a foamy surface;
3) Romberg with eyes closed and firm surface;
4) Romberg with closed eyes and foamy surface;
5) Sensitized Romberg with eyes open and firm surface;
6) Sensitized Romberg with eyes open and foamy surface;
7) Sensitized Romberg with eyes closed and firm surface;
8) Sensitized Romberg with eyes closed and firm surface;
9) Unipodal support with eyes open and firm surface;
10) Unipodal support with eyes open and foamy surface;
11) Unipodal support with eyes closed and firm surface;
12) Unipodal support with eyes closed and foamy surface.

The Romberg test was carried out in the orthostatic position with the arms loose along the body. The Sensitized Romberg was carried out with the feet aligned one in front of the other and the arms loose along the body. In the unipodal support test the patients remained standing up, with only one foot supporting them on the ground^23^. The foot chosen for the ground support in the unipodal support test was the dominant foot. This was established asking the patients with which foot they would preferably kick a ball should it be rolled to them^24^.

The patients underwent the tests wearing comfortable clothes, barefoot and wearing glasses when necessary. They were closely supervised by an examiner, in order to guarantee safety and physical integrity in cases of unbalance. In order to execute the tests, there was no practice. The examiner showed the patients each one of the tasks, in the many proposed sensorial conditions, before holding the test.

When the tests were performed with eyes closed, we used a blindfold covering the eyes and for foam tests we used a pillow of density 28, with sizes 40 × 40 cm.

We had three attempts for each one of the balance tests. Patient performance on the balance tests was classified as YES and NO. We gave it a YES answer when the participant was able to keep himself up standing, without altering the support base for 30 seconds in at least 2 of the 3 attempts. We gave it a NO answer when the patient was unable to maintain the orthostatic position, without changing support base for 30 seconds in 2 or more attempts.

## RESULTS

The present research studied over 100 patients with BPPV diagnosis based on clinical history and positional nystagmus in the Dix-Hallpike diagnostic test. Patient age varied between 17 and 88 years; 74 were females and 26 males.

After SRM, 96% of the patients had resolution of the nystagmus and vertigo, characteristic of BPPV. In average, we needed 1.27 SRMs (from 1 to 3) in order to resolve positional vertigo and nystagmus. In 4% of the patients, positional vertigo and nystagmus were not suppressed with SRM during one year, thus the BPPV was deemed persistent.

Of the 96 patients who had BPPV symptoms cleared up after SRM, 26 returned to the ward with typical BPPV symptoms followed by positional nystagmus in the Dix-Hallpike test during one year. In BPPV recurrence we also needed 1.27 SRM (from 1 to 2) to abolish positional nystagmus and vertigo.

Three of the four patients with BPPV were included in the APVR protocol. One patient developed Alzheimer and left the treatment.

We noticed a reduction in the dizziness analogue score scale (Chart 1) as well as in the Brazilian DHI (Chart 2) after APVR treatment in the three patients with persistent BPPV.

**Chart 1:** Scores in the dizziness analogue scale before and after the aquatic physical therapy protocol for Vestibular Rehabilitation in 3 patients with persistent Benign Paroxysmal Positional Vertigo.

	Before APVR	After APVR
Patient C.S.A	7	4
Patient P.O.	6	1
Patient J.C.A	5	2

Legend: APVR -Aquatic physical therapy for vestibular rehabilitation.

**Chart 2:** Scores in physical, functional and emotional aspects and total score of the application of the Brazilian Dizziness Handicap Inventory questionnaire before and after the aquatic physical therapy protocol for vestibular rehabilitation in 3 patients with persistent Benign Paroxysmal Positional Vertigo.

DHI Scales	Physical		Emotional		Functional		Total	
	Before APVR	After APVR	Before APVR	After APVR	Before APVR	After APVR	Before APVR	After APVR
Patient C.S.A.	26	24	20	14	24	6	70	44
Patient P.O.	22	4	10	0	12	2	44	6
Patient J.C.A.	18	4	24	18	18	8	60	30

Legend: APVR - Aquatic Physical Therapy for vestibular rehabilitation

DHI - Dizziness Handicap Inventory

We noticed improvements in at least three balance sensorial conditions in the three patients with persistent BPPV in our study, as shown in chart 3.

**Chart 3:** Results from balance evaluation of the 3 patients with persistent Benign Paroxysmal Positional Vertigo treated with the Vestibular Rehabilitation Aquatic Physical Therapy protocol.

		Patient C.S.A	Patient P.O	Patient J.C.A
RO/ OA/ SF	Before APVR	Yes	Yes	Yes
	After APVR	Yes	Yes	Yes
RO/ OA/ SE	Before APVR	Yes	Yes	Yes
	After APVR	Yes	Yes	Yes
RO/ OF/ SF	Before APVR	No	Yes	Yes
	After APVR	Yes	Yes	Yes
RO/ OF/ SE	Before APVR	No	No	Yes
	After APVR	Yes	Yes	Yes
ROS/ OA/ SF	Before APVR	Yes	Yes	No
	After APVR	Yes	Yes	Yes
ROS/ OA/ SE	Before APVR	Yes	No	No
	After APVR	Yes	Yes	Yes
ROS/ OF/ SF	Before APVR	No	No	No
	After APVR	Yes	Yes	Yes
ROS/ OF/ SE	Before APVR	No	No	No
	After APVR	No	No	Yes
AU/ OA/ SF	Before APVR	Yes	Yes	Yes
	After APVR	Yes	Yes	Yes
AU/ OA/ SE	Before APVR	No	No	No
	After APVR	Yes	No	No
AU/ OF/ SF	Before APVR	No	No	No
	After APVR	No	No	No
AU/ OF/ SE	Before APVR	No	No	No
	After APVR	No	No	No

Legend:

APVR- Aquatic Physical Therapy for Vestibular Rehabilitation

RO- Romberg

ROS- Sensitized

AU- Unipodal support

OA- Eyes open

OF- Eyes closed

SF - Firm surface

SE - Foamy surface

## DISCUSSION

BPPV is considered the most common cause of labyrinthine dizziness, especially among the elderly, causing quality of life loss, restrictions in social and domestic activities and relevant risk of falls 25.

In our sample, females predominated (74%) with results in agreement with the literature surveyed^17^, ^25^, ^26^. Hormonal alterations more commonly found in women, could favor the highest occurrence of BPPV^26^.

The age of patients with BPPV in the present study varied between 17 to 83 years, variation similar to the one found in the studies by Macias et al.^27^ in which age varied between 20 and 93 years.

We obtained a therapy success rate of 96% using the Epley and Semont maneuvers for the vertical canals and the Lempert maneuver for the horizontal canals with a varied number of maneuvers. Similar results were described by the authors of the maneuvers, presenting rates above 90%^3^, ^4^, ^20^.

Atlas, Parnes28 described three forms of BPPV according to the time of clinical evolution and recurrence pattern:

1) self-limited,
2) recurrent or
3) persistent.

In the self-limited form, the symptoms disappear in weeks or months. In the recurrent form patients have vertigo spells interspersed by asymptomatic intervals. In the persistent form, symptoms do not resolve in a period of at least one year^2^, ^28^. In our series we changed the name “Self-Limited” to “Non-Recurrent”, since the term self-limited may encompass the cases which had spontaneous recovery, regardless of having been submitted to SRM.

Most of the patients in this study had the non-recorrent form of the BPPV (70%). Recurrence rate was of 26% in one year of follow up. The recurrent BPPV was described by some authors with variable results. Thus, while Semont^3^ recognized it in 4.22% of his patients, Baloh^29^mentioned a recurrence rate of 50%. Macias et al.^27^in a prospective study described 13.5% of BPPV symptoms recurrence after six months of SRM. Helminski et al.30 found 43% of BPPV recurrence after SRM, without statistical significance among the patients who did or not the Brandt-Daroff exercises daily as a means to prevent recurrence. This variability in results among the authors can be explained by the difference in time and the mode of patient follow up. We believe that the longer the follow up time of patients, the greater is the proportion of recurrent cases. We stress that in the current study, only the cases in which positional nystagmus were proven by the examiner were considered recurrent.

Four patients (4%) were classified as having persistent BPPV after one year of follow up without symptom remission, despite SRM treatment. According to Gross et al.31, the association between BPPV and Meniere's disease may render BPPV untreatable or persistent. Hall et al.^32^ postulated that persistent BPPV resistant to treatment must have a pathophysiological mechanism different from the non-recurrent forms. Despite SRM failure and BPPV persistence, the following mechanisms may be involved:

1) thinning and weakening of the mucopolyssacaride gelatinous layer on the utriculus's otolythical membrane^33^;
2) intralabyrinthine fibrosis or ossification involving the endolymphatic space^33^;
3) utricular macula lesion or lesion involving the membranous labyrinth^27^;
4) increase in endolymphatic calcium content^34^;
5) cupula lithiasis^35^;
6) endolymphatic hydrops^26^;
7) statocone adherense to the membranous labyrinth^19^;
8) vestibular atelectasia^36^.

Schratzenstaller et al.^37^ found evidence of filling deffects in the semicircular canal seen on a labyrinth MRI of five patients who suffered of untreatable BPPV.

Patients with persistent BPPV in the present investigation had clinical improvement in relation to the impairment dizziness caused to their quality of life, to body balance and the very self-perception of dizziness intensity after being submitted to the APVR protocol, satisfactorily responding to non-surgical treatment (APVR), contrary to results found by Gross et al.^31^, which considered BPPV persistent form as untreatable.

Gabilan^15^ noticed that the APVR protocol proved efficient to improve quality of life, dizziness intensity and body balance in patients with Unilateral Defficitary Peripheral Vestibular Syndrome.

Norré, Beckers^38^ also noticed that habituation exercises are efficient in stimulating vestibular neuroplasticicy in patients who suffered from BPPV. These authors noticed that the results from these exercises are similar to those achieved in the Semont maneuver after 6 weeks of follow up. However, they did not reveal the rate of recurrence and persistence of BPPV in their patients.

Many factors may have contributed to the improvement in these patients. These factors may be didactically broken down into aquatic factors associated to the physical properties of water and the physiological consequences that immersion in water causes to the human body and adaptive factors triggered directly by rehabilitation exercises, stimulating vestibular neuroplasticity.

The thrust that a body immerse in water suffers reduces gravitational stress to the muscles and joints, especially in the lower limbs^39^, which can reduce the sensorial information coming from these joint receptors^40^. The reduction in proprioceptive information creates a sensorial conflict and it can stimulate the systems involved in body balance, causing adaptations to the central processing of this information, motor adjustments and postural corrections. The support property provided by water allows the patient to be more independent in performing his/her activities and provides more time to balance oneself when there is risk of falling.

The practice of exercises in water facilitates vestibular adaptation stimulation, because in order to perform any movement in the water there is the need to start from a stable initial position, requiring balance reactions from the individual in order to maintain body symmetry^41^. The effects of water whirling may also have demanded more from balance and coordination of our patients and, frequently, are used as a resource to develop such skills in aquatic physical therapy exercises, according to Campion^42^.

Patient's visual stimulation is fundamental to recover eyesight stabilization, according to Herdman^43^. The effect of refraction on water causes distortions in member position and the correct posture of the individual in the vertical stance^44^ and may have participated as a stimulant in vestibular compensation mechanisms.

The stimulus created by warm water in the entire submerse body surface in patients with vestibular disorders may have contributed to the favorable therapeutic results as it facilitates the practice of VR exercises, because according to Suomi, Koceja^45^, water in these conditions improve blood circulation and causes muscle relaxation.

Moreover, eyesight deprivation and impairment would increase the sensorial challenge in maintaining body balance. Flotation equipment and the use of stairs with different heights were added to the exercises, and increased the degree of difficulties to maintain body alignment and stability thresholds, stimulating the systems responsible for body balance. Also emphasized is the stimulation of balance control and adjustment in the water, with the performance of motor activities which encompass postural transfers, gait and repeated head movements. One of our goals with VR is to increase static and dynamic postural stability in situations of sensorial conflict^46^. When the somatosensory information and, in a smaller scale, visual is not available or is distorted, vestibular information take on a predominant function for postural control^47^. Activities with balls, similar to the advocated by Ganança et al.8 provides visual clues associated with head movement in order to stimulate the vestibular-ocular reflex.

Horak, Shupert^47^ noticed that the ankle, hip and step strategies are important for the recovery of body balance in the thresholds of body stability and patients with vestibular deficit have a greater difficulty to use the hip strategy. In the APVR protocol, the hip strategy is required in many stages, with the aim of improving movements in this region, improve this type of balance reaction and, concurrently, make up possible postural compensations stemming from the loss of balance. Floating device use requires the individual to be always in the vertical position, constituting a feedback when posture is asymmetrical. Trunk symmetry allows for proper limb performance in the motor coordination tasks promoted in activities with balls, both with the patient seating down as well as standing up.

In treatment planning for these patients with BPPV, it is important to recognize its recurrence and duration patterns. SRM vocation is to shorten symptoms duration during a BPPV spell, being it recurrent or not. In the persistent form, we can choose other treatment modes such as rehabilitation exercises, vestibular suppression drugs, and chemical destruction of the labyrinth or surgical procedures.

Our study pointed out that the use of APVR can be useful in the clinical improvement of patients with persistent BPPV. New studies must be carried out with larger samples of patients with persistent BPPV and control group comparison so that we may establish the efficacy of this treatment method in this population of people with vestibular disorders.

## CONCLUSION

In one year of clinical follow up after positional vertigo and nystagmus resolution by means of SRM, BPPV was not recurrent in 70% of the patients, recurrent in 26% and persistent in 4% of them.
